# Robust nasality representation learning for cleft palate-related velopharyngeal dysfunction screening in real-world settings

**DOI:** 10.3389/fdgth.2026.1833087

**Published:** 2026-07-16

**Authors:** Weixin Liu, Bowen Qu, Amy Stone, Maria Powell, Shama Dufresne, Stephane Braun, Izabela Galdyn, Michael Golinko, Bradley Malin, Zhijun Yin, Matthew E. Pontell

**Affiliations:** 1 Department of Electrical and Computer Engineering, Vanderbilt University, Nashville, TN, United States; 2 Department of Computer Science, Vanderbilt University, Nashville, TN, United States; 3 Department of Otolaryngology—Head and Neck Surgery, Vanderbilt University Medical Center, Nashville, TN, United States; 4 School of Medicine, Vanderbilt University, Nashville, TN, United States; 5 Department of Plastic Surgery, Vanderbilt University Medical Center, Nashville, TN, United States; 6 Division of Pediatric Plastic Surgery, Monroe Carell Jr. Children's Hospital, Nashville, TN, United States; 7 Department of Biomedical Informatics, Vanderbilt University Medical Center, Nashville, TN, United States; 8 Department of Biostatistics, Vanderbilt University Medical Center, Nashville, TN, United States

**Keywords:** cleft palate, digital screening, domain shift, hypernasality, mobile health, nasality representation, supervised contrastive learning, velopharyngeal dysfunction

## Abstract

**Background:**

Velopharyngeal dysfunction (VPD) is an impaired ability to achieve adequate velopharyngeal closure during speech, often resulting in hypernasality and reduced intelligibility. VPD screening and diagnosis require specialized expertise and controlled recording conditions, limiting scalable access outside high-income countries.

Key challenge: Speech-based machine learning models can perform extremely well under standardized clinical recording conditions. However, performance often deteriorates when deployed on consumer devices (e.g., phones or tablets) and in uncontrolled acoustic environments. This degradation is largely driven by *domain shift* arising from differences in recording conditions (e.g., device and channel characteristics, background noise, and room acoustics), which can cause models to rely on spurious recording artifacts rather than pathology-relevant cues.

**Methods:**

This study introduces a two-stage framework to improve robustness under realistic recording scenarios. *Nasality representation pre-training* employs a nasality-focused representation via supervised contrastive learning (SupCon) using an auxiliary dataset with phoneme alignments to form oral-context versus nasal-context supervision. During *Frozen-encoder VPD screening*, the encoder is frozen to perform VPD screening using lightweight classifiers on 0.5-second chunks with probability aggregation to produce recording-level decisions using a fixed decision threshold. Here, *in-domain* refers to standardized clinical recordings used for model development, and *out-of-domain* refers to heterogeneous public Internet recordings collected under uncontrolled conditions and evaluated without any adaptation. The proposed method is then compared against prior-study baselines, including MFCC features and large pretrained speech representations, using the same evaluation protocol.

**Results:**

On the primary in-domain subject-disjoint held-out split of 82 subjects (60 train/22 test; 345 training recordings; 131 test recordings; multiple recordings per subject), the proposed approach reached ceiling recording-level screening performance under this standardized clinical protocol (macro-F1 = 1.000, accuracy = 1.000). To assess sensitivity to this fixed split, an additional subject-level nested 5-fold cross-validation analysis was performed on the full in-domain cohort (82 subjects, 476 recordings), with the encoder frozen and only the second-stage classifiers retrained; the best mean performance was obtained with SVM (macro-F1 = 0*.*981 ± 0*.*022, accuracy = 0*.*985 ± 0*.*016). On a separate out-of-domain set of 131 public Internet recordings, large pretrained speech representations degrade substantially, and MFCC is the strongest baseline (macro-F1 = 0.612, accuracy = 0.641). The proposed method achieves the best overall out-of-domain performance (macro-F1 = 0.679, accuracy = 0.695), improving over the strongest baseline by +0.067 macro-F1 and +0.054 accuracy (point-estimate improvements) under the same evaluation protocol and fixed threshold.

**Conclusion:**

Learning a nasality-focused representation prior to clinical classification can reduce sensitivity to recording artifacts and improve robustness when moving from the laboratory to real-world audio recording scenarios. This design supports practical deployment of VPD screening and motivates domain-robust evaluation protocols for deployable speech-based digital health tools.

## Introduction

Velopharyngeal dysfunction (VPD) arises from inadequate function of the velopharyngeal port during speech, which allows for abnormal air coupling between the oral and nasal cavities. VPD frequently presents with hypernasality and impaired speech intelligibility, both of which negatively impact communication, feeding, and psychosocial function (1). The most common manifestation of VPD is velopharyngeal insufficiency (VPI), which often results from velopharyngeal dysfunction in the setting of a cleft palate (2). In patients with cleft palate, VPI rates can exceed 30% and are likely much higher worldwide (3). Diagnosis and management of VPD require specialized speech-language pathologists (SLPs) who are often members of multidisciplinary cleft care teams, and evaluations are often conducted in standardized acoustic recording conditions. These resources are often not available in low- and middle-income countries (LMICs), thereby creating a barrier to timely diagnosis and management (4).

**Table 1:** Key hyperparameters for nasality supervised contrastive (SupCon) pre-training.

Component	Setting
Backbone	Wav2Vec2-Large-960 h (local checkpoint), last-layer fusion
Input sampling rate	16 kHz
Segment duration	0.20 s (3,200 samples), center crop/zero pad
Embedding dimension	256
Layer fusion	last *K* = 4 layers, learnable softmax weights
Unfreezing	last *N* = 4 transformer layers + encoder layer norm
Loss	vowel-restricted supervised contrastive loss
Temperature	*τ* = 0*.*07
Optimizer	AdamW
Learning rates	head: 3 × 10^−4^; backbone: 3 × 10^−5^
Weight decay	0.01
Gradient clipping	max norm 5.0
Precision	AMP bfloat16
Epochs/early stop	up to 20; patience 6 (monitor: validation pairwise accuracy; SupCon only)
Validation split	10% random split; seed = 42
Batch size	auto-selected from {2048,4096,6144,8192}

In an attempt to augment the reach of existing SLPs, teams have begun to explore the use of machine learning models to automatically detect the presence of VPD from acoustic samples (2, 4–6). To ensure that models are clinically translatable, a scalable VPD screening tool must be deployable in the field and operate on consumer devices in everyday acoustic environments. However, clinical deployment introduces significant variation in acoustic setting, microphone frequency response, channel compression, background noise, reverberation, and speaking style, amongst many other factors. Such factors induce domain shift between standardized clinical recordings used for model development and real-world audio encountered during deployment (6–8). A central challenge is that models trained in-domain can inadvertently focus on spurious correlations tied to recording conditions (e.g., device-specific spectral coloration or background noise patterns), achieving near-ceiling performance in controlled tests yet failing to generalize under realistic variability. This phenomenon is widely recognized as “shortcut learning” in medical AI, where models latch onto non-pathological artifacts rather than actual biological signals (9, 10).

Recent literature on automated VPD, cleft palate speech, and voice-disorder assessment has expanded rapidly. A recent systematic review of artificial intelligence and machine learning models for cleft-related VPD screening highlights the growing interest in automated acoustic screening tools while also emphasizing persistent challenges in dataset size, clinical heterogeneity, and external validation (11). Clinical studies have explored automatic VPD detection from speech samples using support vector machines, neural networks, and large pretrained speech models (2, 4, 5). Related work has investigated objective hypernasality or nasality estimation using deep learning, MFCC-based models, ASR-derived representations, nasal/oral acoustic channels, and more recent multi-task or enhancement-based approaches for cleft palate speech assessment (12–16). In the broader voice AI literature, recent reviews and deployment-oriented studies have similarly emphasized the need for clinically meaningful labels, robust model development, standardized classification frameworks, and careful translation of voice biomarkers from research settings into real-world use (17–22).

In parallel, large self-supervised and weakly supervised speech representations (e.g., Wav2Vec2, HuBERT, data2vec, Whisper encoders) have enabled strong performance on small clinical datasets by leveraging broad pretraining (23–26). However, recent out-of-domain validation and domain-adaptive self-supervised learning studies show that high in-domain accuracy does not guarantee deployable robustness when models are evaluated on recordings collected under different devices, channels, acoustic environments, and speaking conditions (6, 27). Thus, a key open problem is not only whether a model can detect VPD under standardized clinical conditions, but whether its learned representation remains clinically meaningful under substantial recording-domain shift.

**Figure 1:**
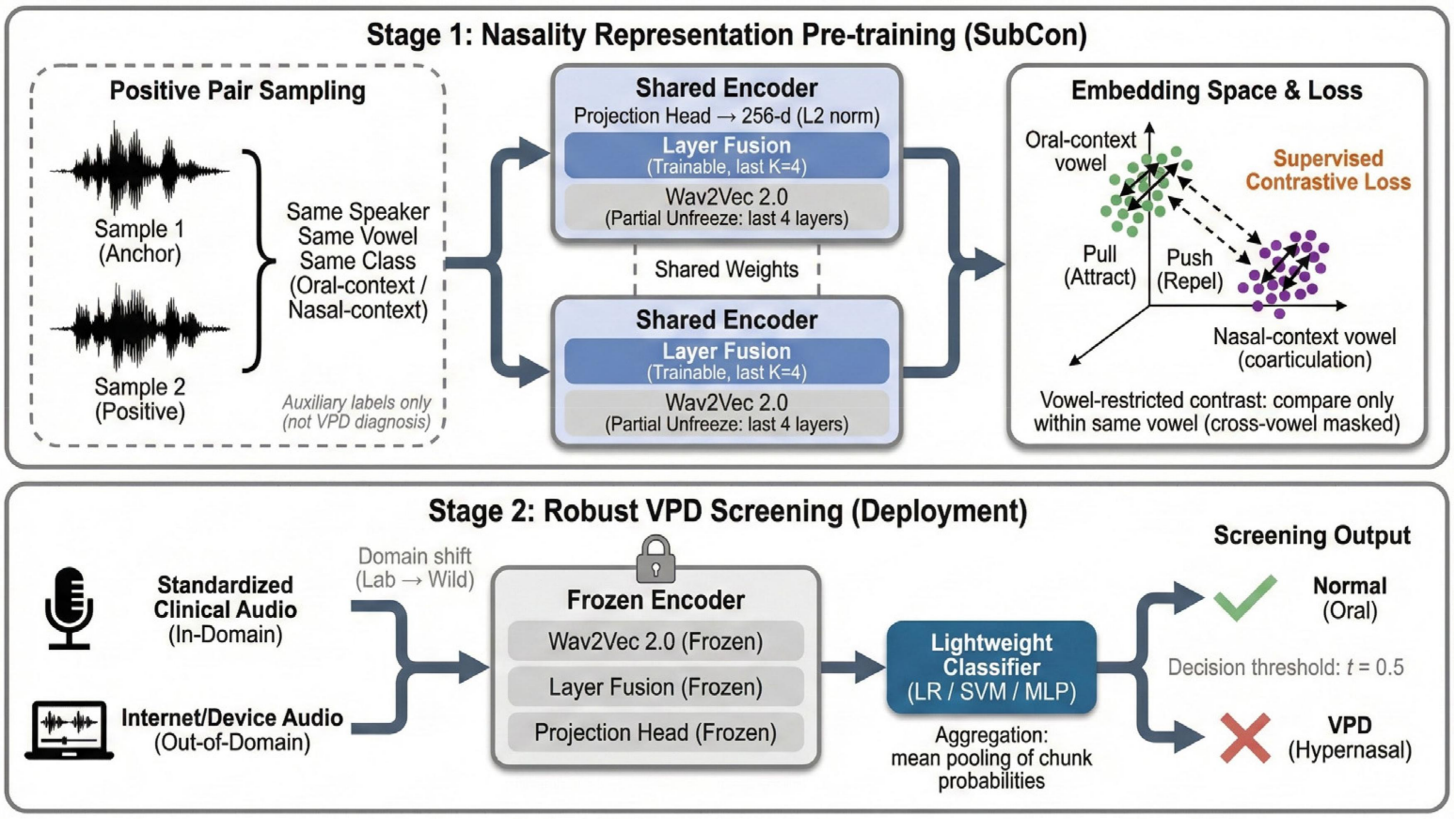
Overview of the proposed two-stage framework. Stage 1: nasality representation pre-training using supervised contrastive learning (SupCon) with positive pairs sampled from the same speaker, same vowel, and same auxiliary class (oral-context vs. nasal-context), while restricting contrastive comparisons to within-vowel pairs to reduce phonetic-content leakage. A Wav2Vec2.0 backbone with trainable layer fusion and a projection head utputs 256-dimensional *ℓ*_2_-normalized embeddings. Stage 2: robust VPD screening under domain shift (lab → wild) using the frozen encoder as a feature extractor on 0.5s chunks, followed by a lightweight classifier (LR/SVM/MLP/XGBoost) and mean aggregation of chunk-level probabilities to produce recording-level screening decisions (in-domain and out-of-domain) using a fixed decision threshold.

This study aims to address this gap by learning a representation that targets the underlying production-related attribute, namely nasality, before training the clinical screening classifier, with the goal of reducing reliance on domain-dependent recording artifacts under cross-device and cross-environment variability (6–8). Such nasality-related cues could be more stable across devices than many channel- or environment-specific artifacts, and thus a representation encouraged to encode nasality distinctions can improve robustness when the recording domain changes (6). To achieve this goal, we use a two-stage framework (Figure 1) to model nasality-related cues. With nasality representation pre-training, supervised contrastive learning (SupCon) is performed on an auxiliary speech corpus with phoneme alignments (28–32). The proposed schema involves constructing an oral-context versus nasal-context supervision signal and applying a sampling strategy that suppresses speaker and phonetic confounds by creating positive pairs from the same speaker and same vowel and by using vowel-restricted contrastive comparisons. With frozen-encoder VPD screening, the learned encoder is frozen and used as a feature extractor for VPD screening with lightweight classifiers, aggregating chunk-level probabilities to yield recording-level screening decisions (in-domain and out-of-domain) using a fixed decision threshold. This design separates representation learning from clinical classification, aiming to improve cross-domain robustness without adaptation to the target domain (6).

This study makes four main contributions. First, it formulates VPD screening as a domain-robust speech representation problem, explicitly evaluating the transition from standardized clinical recordings to heterogeneous real-world Internet audio. Second, it introduces a nasality-focused supervised contrastive pretraining strategy that uses phoneme-alignment-derived oral-context versus nasal-context supervision while controlling speaker and vowel confounds during pair construction. Third, it develops a deployment-friendly frozen-encoder screening pipeline that combines the learned nasality representation with lightweight downstream classifiers and simple recording-level probability aggregation. Fourth, it provides a comprehensive evaluation against classical acoustic features and large pretrained speech representations under the same in-domain and out-of-domain protocols, including additional subject-level cross-validation and clinical target-task representation visualizations.

## Materials and methods

### Datasets and preprocessing

#### Clinical in-domain cohort

The in-domain cohort of 82 patients was collected under standardized acoustic conditions in a clinical setting. Clinical labels were assigned based on *human* specialist speech-language pathology (SLP) perceptual speech evaluation as part of routine clinical assessment, supported by instrumental assessments such as videonasoendoscopy (VNE) and/or nasometry when indicated. To strengthen label validity, all VPD case and control labels used in this study were independently reviewed by two cleft-specialized certified speech-language pathologists (CCC-SLPs). Inter-rater agreement for the binary case/control cohort was perfect (Cohen's *κ* = 1*.*0). The VPD case cohort comprised 44 patients with clinically significant VPD, whereas 38 control patients were identified as having adequate velopharyngeal function based on specialist SLP evaluation. Cohort definition and cohort partitioning followed our established protocol for automated VPD detection and out-of-domain (OOD) validation (4, 6). All recordings were resampled to 16,000 Hz and converted to mono. Each audio file was segmented into multiple non-overlapping 0.5-second chunks. For files shorter than 0.5 s (and residual segments shorter than 0.5 s), we applied repeating padding by tiling the available audio content until reaching the 0.5-second target length (instead of zero-padding) to better preserve short-utterance acoustic characteristics. Each 0.5-second segment was treated as a modeling unit for feature extraction and chunk-level inference.

#### Out-of-domain cohort

To simulate real-world deployment under domain shift, we relied upon an OOD test set constructed from publicly available Internet sources. OOD control-group recordings were sourced from the Centers for Disease Control and Prevention and the Eastern Ontario Health Unit, and OOD case/control labeling and dataset curation followed the same protocol described in a prior OOD validation study (6). The resulting OOD set contains 131 recordings (70 controls, 61 VPD cases). These public sources do not provide reliable speaker identifiers or comprehensive metadata, so each recording is treated as an independent evaluation unit and *recording-level* performance is reported on the OOD set. Models trained on the in-domain cohort were evaluated directly on the OOD recordings *without any retraining, fine-tuning, or calibration* to quantify robustness under domain shift, consistent with prior practice in this clinical context (6). The same procedure used in the clinical in-domain dataset to sample and segment recordings was used for the OOD dataset.

#### Nasality pre-training corpus

To learn a device- and content-robust representation of nasality prior to clinical classification, auxiliary pre-training on the *Librispeech Alignments* dataset was performed (30, 32). This dataset is derived from the LibriSpeech corpus (29) and provides 16kHz read English speech with word- and phoneme-level alignments generated by the Montreal Forced Aligner (MFA) (31). This publicly available alignment resource was introduced in prior work on speech model pre-training for end-to-end spoken language understanding (33). The alignments include phoneme boundaries, enabling time-localized extraction of vowel-centered segments. Using the phoneme-level alignment, we extracted short, vowel-centered acoustic segments from each utterance. Each segment is indexed by a *(vowel, speaker)* key inferred from the filename and metadata. This key was used to construct within-speaker and within-vowel comparisons to minimize confounds from speaker identity and vowel content.

An auxiliary binary supervision signal was constructed for contrastive learning using a rule-based labeling procedure derived from phoneme alignments. Using the MFA-provided ARPAbet phoneme boundaries in the LibriSpeech Alignments dataset, each vowel segment was extracted and assigned a context-based label using its immediate left and right neighboring consonants. Vowels flanked by two oral consonants (C–V–C, where C denotes a non-nasal consonant) were assigned to the oral core set, while vowels flanked by two nasal consonants [N–V–N; *N* ∈ (M, N, NG)] were assigned to the nasal strong set. Mixed contexts (C–V–N or N–V–C) were treated as nasal weak and excluded from SupCon training to reduce label noise from partial coarticulation. As basic quality control, we skipped utterances with missing audio bytes, required a 16 kHz sampling rate, and ignored vowels at utterance boundaries where a full left/right context is unavailable. The speaker identity used for same-speaker sampling was inferred from the utterance ID prefix (e.g., 6415 in 6415-116629-0034). These auxiliary labels are used *only* for representation pre-training and are never used as clinical VPD diagnosis labels. All extracted segments were resampled to 16kHz, converted to mono, and standardized to a fixed duration of 0.20s (3,200 samples) via center cropping (if longer) or zero padding (if shorter).

### Supervised contrastive learning for nasality representation

#### Pair construction and vowel-restricted supervised contrastive objective

A nasality encoder was pre-trained using a supervised contrastive learning (SupCon) process (28). This process extends the foundational self-supervised SimCLR framework (34) by leveraging label information to pull same-class samples together while pushing away opposite-class samples. A sampling strategy specifically designed to suppress speaker and phonetic confounds was employed. Each training item produces two “views” (**x**_1_*,***x**_2_) sampled from two *different* segments belonging to the same class [*y* ∈ (0*,*1)], the same vowel, and the same speaker. This controlled pairing strategy was chosen because the auxiliary oral/nasal labels are derived from phoneme-context rules rather than direct clinical nasality measurements. Under such proxy supervision, segments with the same auxiliary label but different speakers or different vowels can differ substantially in speaker anatomy, pitch, formant structure, vowel-dependent resonance, speaking style, and coarticulatory strength. Treating these highly heterogeneous samples as positives may introduce false-positive contrastive pairs and force invariance to acoustic variation that is not reliably separable from the proxy nasality label. Therefore, same-speaker and same-vowel positive pairing was used as a conservative control-variable design to emphasize local oral-context versus nasal-context differences while suppressing speaker and phonetic-content confounds.

A vowel “*v*” is sampled from the set of vowels that have at least two oral segments and at least two nasal segments. The class label “*y*” is then sampled with equal probability. Finally, we sample two distinct files from the corresponding bucket indexed by key (*v,*speaker):

$$(x1,x2)∼D_{y,v,}\mathrm{speaker},x1\neq x2.$$

To further reduce phonetic content leakage, the contrastive objective is restricted to compare embeddings within the same vowel only. Cross-vowel pairs are excluded from the denominator in the contrastive softmax. In each training step, we sample a mini-batch of *B* paired views, and the encoder outputs embeddings $\{z_{i}^{\left(1\right)},z_{i}^{\left(2\right)}\}\underset{i=1}{B},$ which we concatenate into a set of 2*B ℓ*_2_-normalized vectors. Let *y_i_* denote the oral/nasal label and *v_i_* the vowel ID. Because the embeddings are *ℓ*_2_-normalized, their dot product corresponds to cosine similarity, providing a scale-invariant measure of similarity commonly used in contrastive learning. Pairwise cosine similarities are computed and scaled by a temperature parameter *τ* to control the sharpness of the contrastive softmax distribution:

$$S_{ij}=\frac{z_{i}^{T}z_{j}}{\tau },\tau =0.07$$

Self-similarities are masked (*i* = *j*) and comparisons are restricted to samples with the same vowel (*v_i_* = *v_j_*). The positive set for anchor *i* consists of all samples *j* ≠ *i* such that *y_j_* = *y_i_* and *v_j_* = *v_i_*. The vowel-restricted SupCon loss is:

$$L=-\frac{1}{2B}\sum_{i=1}^{2B}\frac{1}{\left|P(i)\right|}\sum_{\;p\in P(i)}\log\frac{\exp\;(S_{ip})}{\sum_{a\in A(i)}\exp\;(S_{ia})},$$

where A(*i*) = {*j* ≠ *i*: *v_j_* = *v_i_*} and P(*i*) ⊂ A(*i*).

#### Encoder architecture: Wav2Vec2 with layer fusion and partial unfreezing

A Wav2Vec2-style transformer encoder is used as the backbone feature extractor (23). The backbone is initialized from a Wav2Vec2-Large-960 h pretrained checkpoint that is stored locally (“wav2vec2-large-960h-local”). The final *K* = 4 hidden layers are then fused with learnable weights, and the pooled representation is projected to a 256-dimensional embedding, where **H**^(*ℓ*)^ ∈ *R^B^* ^×^ *^S^* ^×^ *^d^* denotes the hidden states from layer *ℓ*. A weighted sum of the final four layers is then computed:

$$H_{\mathrm{fused}}=\sum_{i=1}^{K}\alpha_{i}H^{\left(L-K+i\right)},\alpha_{i}=\frac{\exp\;(w_{i})}{\sum_{\;j=1}^{K}\exp\;(w_{j})},$$

where {*w_i_*} are learnable parameters and *L* is the total number of transformer layers. Mean pooling is then applied over the sequence dimension:

$$h=\mathrm{MeanPool}(H_{\mathrm{fused}})\in R^{B}\times d,$$

followed by a two-layer MLP projection head to produce a 256-dimensional embedding:

$$z=Normalize\;(BN(MLP_{\left(h\right)}))\in R^{B\times 256}$$

Embeddings are *ℓ*_2_-normalized. Batch normalization is applied during training when the batch size is greater than 1. To balance adaptation and stability, the backbone is frozen except for the last *N* = 4 transformer layers (and the encoder layer normalization), while always training the layer-fusion weights and the projection head. In summary, the first *L*−4 transformer layers are kept fixed and only the last four layers are fine-tuned during SupCon pre-training.

#### Training details

The model is then trained using AdamW with weight decay 0.01 and two parameter groups to implement layer-wise learning rates: (i) fusion/projection head parameters at 3 × 10^−4^, and (ii) unfrozen backbone layers at 3 × 10^−5^. Gradients were clipped to a maximum norm of 5.0, mixed precision training used bfloat16, and training ran up to 20 epochs with early stopping (patience = 6) based on the validation monitor below. A random 10% split of the auxiliary dataset was used as validation (seed = 42). Batch size was auto-selected from {2048, 4096, 6144, 8192} to maximize throughput under GPU memory. Unless otherwise specified, all Wav2Vec2 backbone weights were initialized from the same Wav2Vec2-Large-960 h pretrained checkpoint and loaded from a locally cached copy for training efficiency.

No additional acoustic degradation augmentation was applied during nasality SupCon pre-training. This choice was made to isolate the effect of the nasality-focused contrastive objective under controlled phoneme-alignment-derived supervision. Because the auxiliary oral/nasal labels are proxy labels derived from local phoneme context rather than direct clinical nasality measurements, aggressive augmentations such as noise injection, reverberation simulation, device/channel filtering, or pitch shifting could alter resonance- and coarticulation-related cues that are central to the oral-context versus nasal-context distinction. Thus, augmentation was not used in the present pre-training stage, while nasality-preserving acoustic augmentation is treated as an important direction for future robustness improvements.

#### Representation quality monitor

The embedding space was evaluated using a pairwise distance discrimination task on the validation split. Under matched vowel and matched speaker, positive pairs are same-class (oral–oral or nasal–nasal) and negative pairs are cross-class (oral–nasal). Given embeddings $z_{1},z_{2}$, the Euclidean distance was computed:

$$d=\;∥z_{1}-z_{2}{∥}_{2}.$$

Representation quality was evaluated using a distance-based pairwise discrimination task under matched speaker and matched vowel. For early stopping, validation pairwise accuracy is computed by selecting a fixed distance threshold on the validation split and applying it consistently across vowels. This monitor is used only to track representation learning during SupCon pre-training; it is not used for the downstream VPD screening classifier, which uses a fixed decision threshold on predicted probabilities.

#### Clinical target-task representation visualization

To assess whether the learned representation was aligned with the downstream clinical screening task, we additionally visualized frozen SupCon nasality embeddings from the in-domain clinical cohort using UMAP. For each subject, 256-dimensional chunk-level embeddings were first extracted from all 0.5second clinical speech chunks using the frozen SupCon nasality encoder. Embeddings from all chunks and recordings belonging to the same subject were then mean-pooled and *ℓ*_2_-normalized to obtain a subject-level representation. To avoid using held-out test subjects during manifold fitting, standardization, PCA, and UMAP were fit using only the in-domain training subjects, and held-out test subjects were subsequently projected into the same two-dimensional space. PCA retained up to 50 components before UMAP. UMAP was computed using cosine distance, *n*_neighbors_ = 15, min dist = 0*.*1, and random seed 42. Clinical labels were used only for visualization and were not used to fit the UMAP projection.

### Lightweight VPD classification

After pre-training, the encoder is frozen and 256-dimensional embeddings are extracted for each 0.5-second chunk in the clinical datasets. Several lightweight classifiers are trained on top of these embeddings using only the in-domain training data: logistic regression (LR), support vector machine (SVM), multilayer perceptron (MLP), and XGBoost (35). These models are computationally efficient and represent complementary decision functions (linear, margin-based, shallow nonlinear, and gradient-boosted trees), allowing a well-performing yet deployment-friendly classifier to be selected via cross-validation without changing the underlying representation. For all classifiers, z-score standardization was fit on the training fold only and then applied to the corresponding validation or test data. The hyperparameters were selected via group-wise cross-validation on the in-domain training split using GroupKFold to prevent subject leakage, where all recordings (and their constituent chunks) from the same subject were kept in the same fold. The selection criterion was chosen to be recording-level macro-F1 averaged across folds, where chunk-level probabilities were first aggregated within each recording. The searched grids were:

- LR: *C* ∈ {0*.*01*,*0*.*1*,*1*,*10} (class-weight balanced).
- SVM: we tuned the kernel type and associated hyperparameters (e.g., *C* for linear; *C* and *γ* for RBF) using cross-validation (class-weight balanced; probability outputs enabled).
- MLP: hidden sizes ∈ {(64),(128*,*64)}, activation ∈ {relu*,*tanh}, and *α* ∈ {10^−4^*,*10^−3^} with early stopping.
- XGBoost: we tuned standard gradient-boosted tree hyperparameters (e.g., max depth, number of estimators, and learning rate) via cross-validation.

For brevity, the SVM results are reported without specifying the kernel in the tables. The best performing SVM configuration was selected by cross-validation. After setting the hyperparameters, each classifier was refit on the full in-domain training split and evaluated on the held-out test split.

### VPD classification baseline models

To ensure a fair and direct comparison with prior work, commonly used baseline feature extractors and classical classifiers are evaluated under the same preprocessing and evaluation protocol. Five feature extraction pipelines are implemented for each 0.5-second chunk:

- MFCC (baseline) (36): 40 MFCC coefficients per chunk, mean-pooled over frames (40-d).
- Wav2Vec2-Large-960 h (frozen) (23): final-layer hidden states mean-pooled over time (1024-d).
- HuBERT-Large (frozen) (24): final-layer hidden states mean-pooled (1024-d).
- Data2Vec-Audio-Large (frozen) (25): final-layer hidden states mean-pooled (1024d).
- Whisper-Large-v2 encoder (frozen) (26): encoder hidden states mean-pooled (1280-d).

Four classifiers were evaluated for each feature type: SVM, logistic regression, MLP, and XGBoost (35) (20 feature-classifier pipelines). For SVM, the kernel and associated hyperparameters were selected through cross-validation. For brevity, SVM results are reported without kernel specification.

### Model evaluation

Recording-level screening performance is reported for both the in-domain clinical cohort and the OOD Internet recordings. Each recording is segmented into non-overlapping 0.5-second chunks and the classifier outputs a chunk-level probability *p*^*_i_* for each chunk. For a recording with *N* chunks, a recording-level probability is computed by mean aggregation. The recording-level probability aggregation and evaluation metrics are defined in Equations 1–3.

$${}^{{\hat{\;p}}_{rec}}=\frac{1}{N}\sum_{i=1}^{N}{}^{\hat{\;p}i}$$ (1)

A fixed decision threshold *t* = 0*.*5 is then applied to obtain the binary screening decision. For the OOD Internet recordings, each recording is treated as an independent evaluation unit and the same aggregation and thresholding procedure is applied.

To prevent subject leakage (i.e., recordings from the same individual appearing in both the training and test sets), all in-domain train/test partitions were defined at the subject level such that no subject was included in both the training and testing sets. In the primary held-out evaluation, we used the original 60-subject training split and 22-subject held-out test split. Hyperparameter selection for this primary evaluation was performed with group-wise cross-validation (GroupKFold) on the in-domain training split, where all recordings and their constituent chunks from the same subject were assigned to the same fold. This design prevents the model from exploiting repeated recordings from the same individual across training and evaluation.

To assess whether the in-domain results were sensitive to the specific fixed 60/22 subject split, we additionally performed a nested 5-fold subject-disjoint cross-validation sensitivity analysis on the full in-domain cohort (82 subjects, 476 recordings). In this analysis, the SupCon nasality encoder was kept frozen, and only the second-stage lightweight classifiers were retrained within each outer fold. The outer folds were generated at the subject level, ensuring that all recordings from the same patient appeared in either the training or test portion of a fold, but not both. Within each outer-training split, classifier hyperparameters were selected using inner subject-disjoint cross-validation. Recording-level predictions were obtained using the same 0.5-second chunking, mean probability aggregation, and fixed decision threshold of *t* = 0*.*5.

It should be noted that this retrospective clinical cohort may exhibit demographic differences between cases and controls (e.g., age and sex distributions), which can act as potential confounders. For the OOD dataset, speaker identities are not provided and cannot be reliably inferred from the public sources. Therefore, speaker-disjoint evaluation cannot be enforced and instead, evaluation is performed directly at the recording level without any retraining, fine-tuning, or calibration.

All metrics are computed at the recording level, and accuracy, macro-precision, macro-recall, and macro-F1 are reported. For each class *c* ∈ {0*,*1} (either control or VPD), precision, recall, and F1 are:

$$Prec_{c}=\frac{TP_{c}}{TP_{c}+FN_{c}},Rec_{c}=\frac{TP_{c}}{TP_{c}+FN_{c}},\mathrm{and}\;F1_{c}=\frac{2\mathrm{Pr}ec_{c}Rec_{c}}{\mathrm{Pr}\;\;ec_{c}+Rec_{c}}$$ (2)

And macro-precision, macro-recall, and macro-F1 are computed as the unweighted mean across classes:

MacroPrec=12∑cPrecc,MacroRec=12∑cRecc,MacroF1=12∑cF1c, (3)

Accuracy is computed as the fraction of correctly classified recordings.

## Results

### Dataset summary

The in-domain cohort consists of 82 subjects and was partitioned into an in-domain 60-subject training set and a 22-subject held-out test set using a subject-disjoint split to prevent subject leakage. The training set comprised 28 controls and 32 VPD cases with 345 recordings, while the held-out test set comprised 10 controls and 12 VPD cases with 131 recordings. In the 60-subject training cohort, the controls were 71.4% female with a mean age of 29*.*6 ± 11*.*8 years, while the VPD cases were 46.9% female with a mean age of 10*.*0 ± 3*.*8 years. In the 22-subject held-out test cohort, the controls were 70.0% female with a mean age of 30*.*8 ± 10*.*2 years, while the VPD cases were 41.7% female with a mean age of 9*.*1 ± 3*.*6 years. Each subject contributed one or more recordings; therefore, although the split is defined at the subject level, model performance is reported at the recording level. All in-domain case/control labels were independently reviewed by two cleft-specialized CCC-SLPs, with perfect inter-rater agreement for the binary cohort labels (Cohen's *κ* = 1*.*0). This dual-SLP verification provides a clinically reliable label reference for evaluating model performance in a setting where inter-rater-reliability-confirmed AI/VPD cohorts remain uncommon.

The OOD set contains 131 recordings (70 controls, 61 VPD cases), collected under largely undocumented recording conditions (device, environment, and channel characteristics), thereby introducing substantial heterogeneity. Because these public sources do not provide reliable speaker identifiers or comprehensive metadata, we treat each recording as an independent evaluation unit and report recording-level performance on the OOD set. The dataset for nasality SupCon pre-training includes 778,110 oral core segments and 42,670 nasal strong segments, with 406,473 nasal weak segments set aside to reduce label noise.

### Nasality representation pre-training validation

Evaluation is performed to determine whether the SupCon pre-training objective yields a meaningful nasality-focused embedding space under controlled comparisons (matched speaker and matched vowel). On the validation split, the nasality encoder achieved the highest validation pairwise accuracy at epoch 19. Using a fixed distance-based decision rule on the validation split to discriminate matched-speaker, matched-vowel positive vs. negative pairs, the pairwise validation accuracy reached 0.724. This validation monitor is used only for SupCon pre-training early stopping and is separate from downstream VPD screening evaluation.

**Figure 2:**
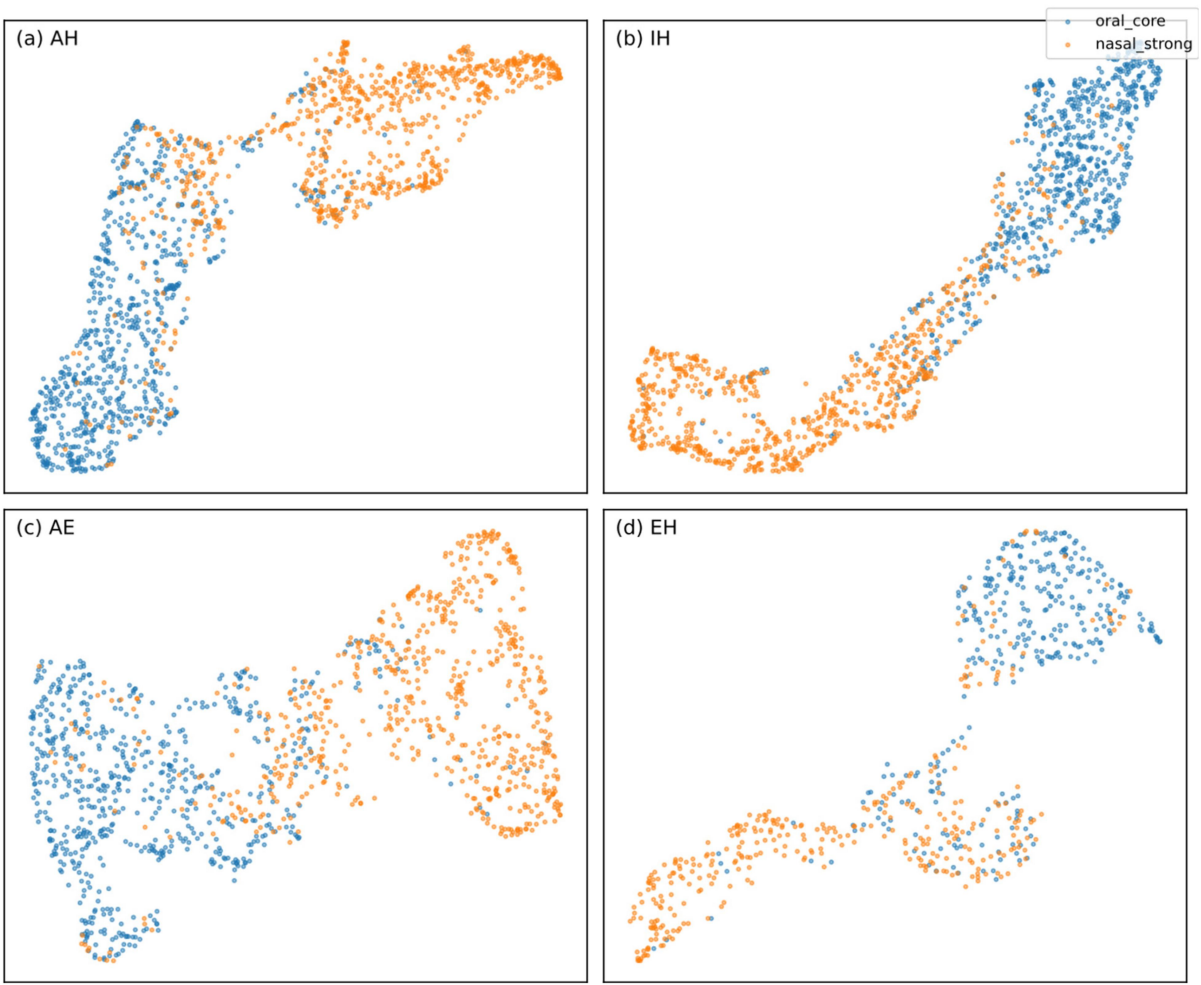
UMAP visualization of SupCon nasality embeddings on the auxiliary validation split. Each panel shows a single vowel with a class-balanced subset of vowel-centered segments (0.20s). Points are colored by the auxiliary nasality context label (oral core vs. nasal strong). Vowel labels follow ARPAbet notation from the forced-alignment annotations.

To qualitatively assess separation in the learned embedding space, validation embeddings are visualized using UMAP for multiple representative vowels (ARPAbet labels; e.g., AH, AE, IH, EH) with class-balanced subsets (Figure 2). We examined whether oral core and nasal strong segments formed distinguishable clusters (or a consistent separation trend) within each vowel, indicating that the embedding captures nasality-related structure beyond vowel identity. Across the examined vowels, oral core and nasal strong segments show partial separation, consistent with the pairwise discrimination results.

**Figure 3:**
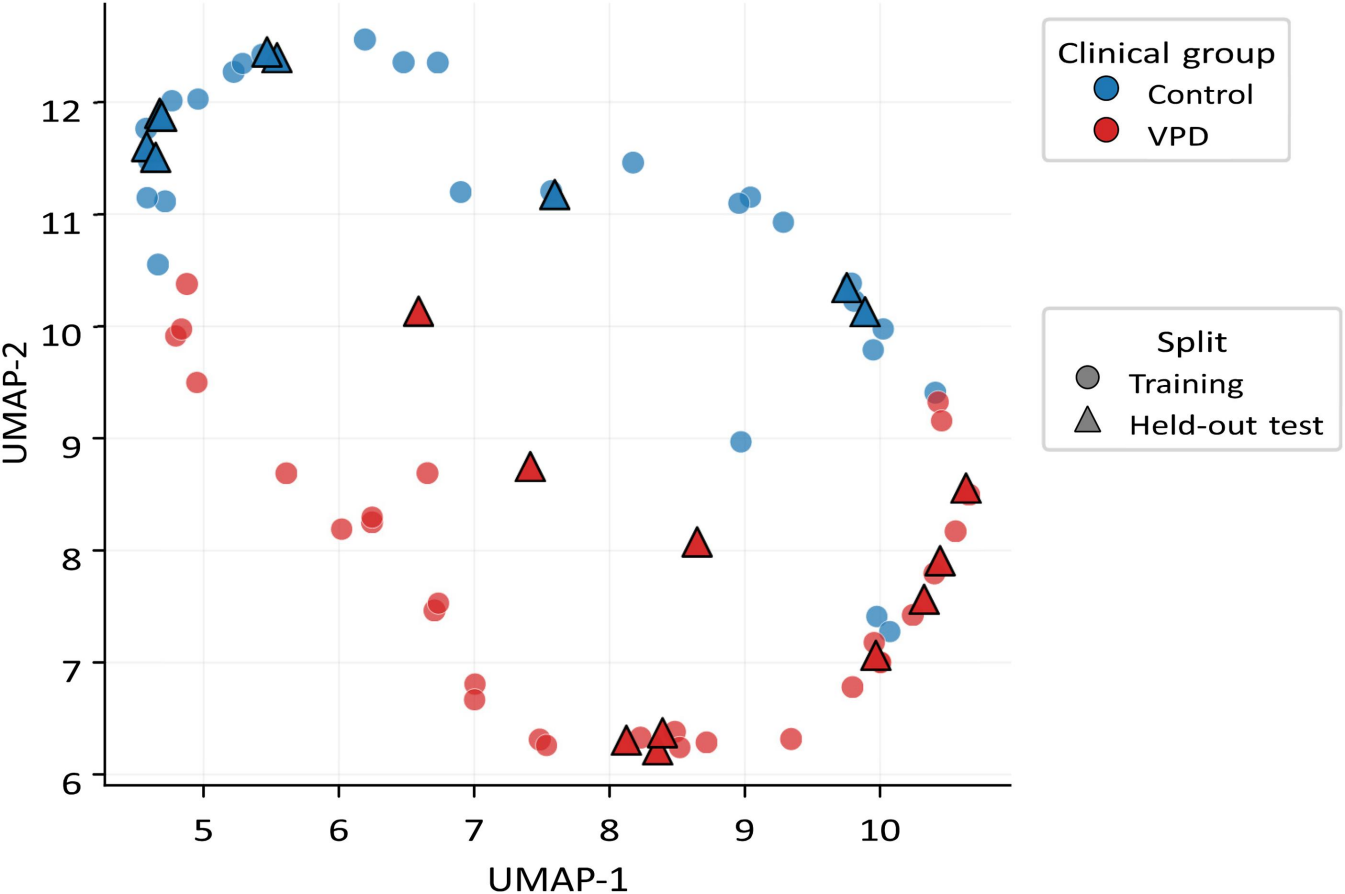
UMAP visualization of subject-level frozen SupCon nasality embeddings from the full in-domain clinical cohort. Each point represents one subject-level embedding obtained by mean-pooling 0.5-second chunk embeddings across all recordings from the same subject followed by *ℓ*_2_ normalization. UMAP was fit on the in-domain training subjects without using clinical labels, and held-out test subjects were projected into the same two-dimensional space. Points are colored by clinical group (control vs. VPD case), and marker style indicates split (training vs. held-out test).

To further evaluate whether the frozen nasality representation aligns with the downstream clinical screening task, we visualized subject-level SupCon nasality embeddings from the full in-domain clinical cohort (Figure 3). Each point represents one subject-level embedding obtained by mean-pooling frozen 256dimensional chunk embeddings across all recordings from the same subject, followed by *ℓ*_2_ normalization. UMAP was fit using only the in-domain training subjects without clinical labels, and held-out test subjects were projected into the same two-dimensional embedding space. The resulting visualization showed partial separation between VPD cases and controls, with held-out test subjects following a similar organization to the training subjects. This qualitative analysis suggests that the frozen nasality representation captures structure relevant to the downstream clinical task, complementing the auxiliary oral-context versus nasal-context proxy visualization.

### Screening performance under in-domain and out-of-domain evaluation

VPD screening performance is evaluated using (i) baseline feature + classifier pipelines from the prior study evaluation protocol (4, 6) and (ii) the proposed SupCon nasality representation with lightweight classifiers. To streamline comparison and emphasize robustness, we report merged tables for the primary in-domain held-out evaluation and OOD evaluation (Tables 2, 4). We additionally report a subject-level nested 5-fold cross-validation sensitivity analysis on the full in-domain cohort to assess the stability of the in-domain findings with respect to the original fixed 60/22 subject split (Table 3).

**Table 2:** Recording-level performance on the in-domain held-out recordings (standardized clinical recordings; 131 recordings from 22 held-out subjects). This table merges (i) all baseline pipelines under the prior study protocol with (ii) the new SupCon nasality representation with lightweight classifiers. Bold highlights the proposed method for ease of comparison.

Feature/Method	Classifier	Accuracy Macro Prec. Macro Rec. Macro F1			
Baselines (prior study protocol)					
Whisper	MLP	1.000	1.000	1.000	1.000
Whisper	XGBoost	1.000	1.000	1.000	1.000
Whisper	SVM	1.000	1.000	1.000	1.000
Whisper	Logistic Regression	1.000	1.000	1.000	1.000
HuBERT	MLP	1.000	1.000	1.000	1.000
HuBERT	XGBoost	1.000	1.000	1.000	1.000
HuBERT	SVM	0.992	0.929	0.996	0.960
Data2Vec	XGBoost	0.992	0.929	0.996	0.960
MFCC	SVM	0.992	0.996	0.917	0.953
MFCC	MLP	0.992	0.996	0.917	0.953
MFCC	Logistic Regression	0.992	0.996	0.917	0.953
Data2Vec	Logistic Regression	0.985	0.875	0.992	0.925
HuBERT	Logistic Regression	0.985	0.875	0.992	0.925
MFCC	XGBoost	0.985	0.913	0.913	0.913
Data2Vec	SVM	0.969	0.800	0.984	0.867
Data2Vec	MLP	0.954	0.750	0.976	0.821
Wav2Vec2	XGBoost	0.946	0.731	0.972	0.801
Wav2Vec2	SVM	0.946	0.731	0.972	0.801
Wav2Vec2	MLP	0.930	0.700	0.963	0.767
Wav2Vec2	Logistic Regression	0.907	0.667	0.951	0.724
Proposed (SupCon nasality representation; 256-d embeddings from 0.5s chunks)					
SupCon Nasality (256-d)	Logistic Regression	1.000	1.000	1.000	1.000
SupCon Nasality (256-d)	SVM	1.000	1.000	1.000	1.000
SupCon Nasality (256-d)	MLP	1.000	1.000	1.000	1.000
SupCon Nasality (256-d)	XGBoost	1.000	1.000	1.000	1.000

**Table 3:** Subject-level nested 5-fold cross-validation sensitivity analysis on the full in-domain cohort. The SupCon nasality encoder was kept frozen, and only the second-stage classifier was retrained within each outer fold. Hyperparameters were selected using inner subject-disjoint cross-validation on the training portion of each outer fold. Metrics were computed at the recording level using chunk-level probability averaging and the fixed decision threshold *t* = 0*.*5. Values are reported as mean ± standard deviation across the five outer folds.

Classifier	Accuracy	Macro Precision	Macro Recall	Macro F1
Logistic Regression	0*.*982 ± 0*.*018	0*.*979 ± 0*.*024	0*.*976 ± 0*.*028	0*.*976 ± 0*.*026
SVM	0*.*985 ± 0*.*016	0*.*982 ± 0*.*021	0*.*981 ± 0*.*023	0*.*981 ± 0*.*022
MLP	0*.*979 ± 0*.*021	0*.*975 ± 0*.*028	0*.*971 ± 0*.*033	0*.*972 ± 0*.*030
XGBoost	0*.*972 ± 0*.*026	0*.*968 ± 0*.*034	0*.*963 ± 0*.*039	0*.*964 ± 0*.*036

**Table 4:** Recording-level performance on the out-of-domain test set (heterogeneous public internet recordings). The table merges (i) all baseline pipelines under the prior study protocol and (ii) the proposed SupCon nasality representation with lightweight classifiers. All metrics use a fixed decision threshold of *t* = 0*.*5.

Feature/Method	Classifier	Accuracy	Macro Prec.	Macro Rec.	Macro F1
Baselines (prior study protocol)					
MFCC	SVM	0.641	0.763	0.663	0.612
MFCC	Logistic Regression	0.603	0.745	0.628	0.560
MFCC	XGBoost	0.550	0.754	0.579	0.473
MFCC	MLP	0.512	0.619	0.540	0.432
Wav2Vec2	MLP	0.512	0.619	0.540	0.432
Wav2Vec2	Logistic Regression	0.504	0.588	0.532	0.427
Data2Vec	SVM	0.504	0.605	0.533	0.419
Data2Vec	MLP	0.489	0.537	0.515	0.417
Data2Vec	Logistic Regression	0.473	0.495	0.498	0.413
Wav2Vec2	SVM	0.504	0.671	0.535	0.402
Data2Vec	XGBoost	0.481	0.527	0.509	0.397
Wav2Vec2	XGBoost	0.489	0.571	0.518	0.393
HuBERT	MLP	0.504	0.742	0.536	0.393
HuBERT	Logistic Regression	0.489	0.738	0.521	0.364
HuBERT	SVM	0.481	0.736	0.514	0.349
HuBERT	XGBoost	0.481	0.736	0.514	0.349
Whisper	Logistic Regression	0.473	0.735	0.507	0.334
Whisper	XGBoost	0.473	0.735	0.507	0.334
Whisper	MLP	0.473	0.735	0.507	0.334
Whisper	SVM	0.466	0.233	0.500	0.318
Proposed (SupCon nasality representation; 256-d embeddings; recording-level aggregation)					
SupCon Nasality (256-d)	MLP	0.695	0.712	0.683	0.679
SupCon Nasality (256-d)	SVM	0.672	0.680	0.661	0.658
SupCon Nasality (256-d)	Logistic Regression	0.664	0.662	0.662	0.662
SupCon Nasality (256-d)	XGBoost	0.655	0.659	0.661	0.658

For the in-domain held-out recordings, several pipelines achieved near-ceiling performance, i.e., mean performance at or near the upper bound observed on this standardized clinical dataset under the current evaluation protocol. Multiple large pretrained speech representations reached 100% accuracy and macro-F1 of 1.000 (e.g., Whisper and HuBERT with MLP/XGBoost), indicating that under controlled recording conditions the screening task is highly separable for these data (Table 2). Using frozen 256-d SupCon nasality embeddings, all evaluated lightweight classifiers (LR/SVM/MLP/XGBoost) also achieved perfect recording-level screening performance (accuracy = 100%, macro-F1 = 1.000). It should be noted that such perfect performance is specific to this in-domain cohort and protocol; it is observed across multiple strong baselines and is consistent with the high separability of the standardized clinical recordings and the recording-level aggregation used in evaluation, rather than implying uniformly perfect performance in more heterogeneous settings.

On the OOD dataset, performance drops substantially across most baseline pipelines, consistent with a strong domain shift between standardized clinical recordings and uncontrolled Internet audio (Table 4). Among the baselines, MFCC + SVM is the strongest (macro-F1 = 0.612; accuracy = 64.1%), while large pretrained speech representations degrade markedly (best macro-F1 among them = 0.432). The proposed SupCon nasality representation achieves the best overall OOD performance, with macro-F1 = 0.679 and accuracy = 69.5% (MLP), improving over the strongest baseline by +0.067 macro-F1 and +5.4 accuracy points under the same evaluation protocol and fixed threshold.

### In-Domain 5-fold cross-validation sensitivity analysis

To address the possibility that the in-domain results were sensitive to the original fixed subject-disjoint train/test split, we performed an additional nested 5-fold cross-validation sensitivity analysis on the full in-domain cohort (82 subjects, 476 recordings). The SupCon nasality encoder was kept frozen, and only the second-stage lightweight classifiers were retrained within each fold. The outer folds were generated at the subject level, ensuring that all recordings from the same patient appeared in either the training or test portion of a fold, but not both. Within each outer-training split, classifier hyperparameters were selected using inner subject-disjoint cross-validation. Recording-level predictions were obtained using the same 0.5-second chunking, mean probability aggregation, and fixed decision threshold of *t* = 0*.*5.

Across the five outer folds, the proposed SupCon nasality representation maintained strong in-domain performance across all lightweight classifiers (Table 3). The best mean performance was obtained with SVM, which achieved an accuracy of 0*.*985 ± 0*.*016 and macro-F1 of 0*.*981 ± 0*.*022. Logistic regression and MLP showed similarly high performance, with macro-F1 values of 0*.*976 ± 0*.*026 and 0*.*972 ± 0*.*030, respectively. These results suggest that the strong in-domain performance is unlikely to be solely attributable to the particular 60/22 subject split used in the primary held-out evaluation. At the same time, the nonzero fold-to-fold variation suggests that performance estimates remain sensitive to cohort composition, which is expected given the modest absolute size of the clinical dataset for machine learning.

## Discussion

This investigation addresses a practical question in speech-based digital health: how can we maintain the performance of a model used to screen for speech pathology when moving from a regulated clinical setting into an unregulated field-testing environment? Motivated by the hypothesis that nasality-related cues may be more stable across recording conditions than many device- and environment-dependent artifacts, this study tested whether learning a nasality-focused representation prior to clinical classification improves robustness under recording domain shift. This hypothesis was evaluated by training models on the in-domain clinical cohort and assessing performance on a separate OOD Internet set without any target-domain retraining, fine-tuning, or calibration under a fixed screening threshold. Under this protocol, the proposed SupCon nasality representation achieved the best OOD performance (macro-F1 = 0.679, accuracy = 0.695), improving over the strongest retained baseline (MFCC + SVM: macro-F1 = 0.612, accuracy = 0.641). In-domain held-out performance was near-ceiling across multiple strong pipelines, and the additional subject-level nested 5-fold cross-validation sensitivity analysis showed consistently strong performance across folds, with the best mean performance obtained by SVM (macro-F1 = 0*.*981 ± 0*.*022, accuracy = 0*.*985 ± 0*.*016). These findings suggest that the strong in-domain performance is unlikely to be solely attributable to the original fixed 60/22 subject split, while also underscoring that high in-domain accuracy does not guarantee deployable robustness under OOD recording conditions (6).

Four specific contributions should be noted. First, this workflow specifically studies VPD screening under substantial domain shift, from standardized clinical recordings to heterogeneous real-world/Internet recordings, using a unified evaluation protocol and a fixed screening threshold. Second, a supervised contrastive pre-training strategy is introduced that learns nasality-sensitive embeddings using oral-context versus nasal-context supervision derived from phoneme alignments, with sampling constraints to reduce speaker and phonetic-content leakage. Third, a two-stage, deployment-friendly pipeline was developed, with a frozen encoder feature extractor, lightweight classifiers, and simple probability aggregation. Fourth, this study provides controlled comparisons against MFCC features and large pretrained speech representations under the same protocol, demonstrating improved robustness in OOD testing. Collectively, these contributions provide evidence consistent with the hypothesis that nasality-focused representation learning can improve robustness under recording-domain shift, and they suggest a practical pathway toward scalable VPD screening on consumer devices where recording conditions are inherently variable and uncontrolled.

An additional strength of this study is the clinical label verification process. All in-domain case/control labels were independently reviewed by two cleft-specialized CCC-SLPs, yielding perfect inter-rater agreement for the binary labels (Cohen's *κ* = 1*.*0). In the context of AI-based VPD screening, where inter-rater-reliability-confirmed cohorts remain uncommon, this label verification increases confidence that model evaluation reflects clinically meaningful VPD status rather than noisy ground-truth labels.

### Representation learning under domain shift

A possible explanation for domain shift is that models trained and tested only on standardized clinical recordings can leverage recording-condition cues that are stable within a given setting (e.g., channel response, compression characteristics, background noise profiles, or room acoustics) but do not reflect pathology. As a result, performance can appear near-ceiling in-domain yet degrade sharply when those recording conditions change in real-world audio (6, 7). In our in-domain cohort, several strong feature + classifier pipelines achieve perfect recording-level performance under the current protocol, suggesting that the standardized clinical recordings are highly separable for this dataset and that recording-level aggregation further amplifies separability. Perfect in-domain testing results can therefore be interpreted as the upper bound for this specific cohort and evaluation setting; however, this performance cannot be implied across non-standardized acoustic settings.

SupCon pre-training provides a more direct mechanism to emphasize nasality-related structure. The oral-context versus nasal-context supervision, together with same-speaker/same-vowel positive pairing and vowel-restricted contrastive comparisons, encourages embeddings to group according to nasality context while suppressing speaker identity and phonetic-content confounds (28). This focus on nasality-related distinctions is consistent with both the improved out-of-domain screening performance observed without any target-domain adaptation and the subject-level clinical UMAP visualization, in which frozen SupCon nasality embeddings showed partial organization by clinical group while heldout test subjects projected into the same representation space as training subjects (6). This interpretation is also aligned with recent clinical voice AI work emphasizing that domain-aware representation learning can be important when voice-based models are transferred across heterogeneous recording conditions (19, 20, 27).

The improved OOD performance of the proposed approach is likely attributable to several complementary factors. First, the representation is pretrained to emphasize nasality-related acoustic structure before exposure to the small clinical VPD dataset, reducing reliance on diagnostic labels that may be entangled with cohort- or recording-specific artifacts. Second, same-speaker and same-vowel contrastive pairing controls major sources of nuisance variability during pre-training, encouraging the encoder to focus on local oral-context versus nasal-context differences rather than speaker identity or broad phonetic content. Third, freezing the encoder and training only lightweight downstream classifiers reduces the risk of overfitting to standardized clinical recording conditions. Finally, applying the same recording-level aggregation and fixed decision threshold across in-domain and OOD evaluation avoids target-domain adaptation, so the observed OOD gains are more directly attributable to the learned representation rather than *post hoc* calibration. Together, these mechanisms provide a plausible explanation for why the proposed nasality representation improves OOD screening performance relative to both MFCC-based baselines and large general-purpose speech representations.

Acoustic data augmentation is an important complementary strategy for improving robustness under real-world recording variability. For example, prior work on robust dysphonic voice embeddings has used data warping with background noise, environmental and device impulse responses, and pitch shifting to improve robustness across corpora and degraded acoustic conditions (37). In the present study, we did not apply acoustic degradation augmentation during nasality SupCon pre-training in order to isolate the contribution of the nasality-focused contrastive objective under controlled proxy supervision. Because the pre-training labels are derived from phoneme context rather than direct clinical nasality measurements, future work should investigate augmentation policies that preserve nasality-relevant resonance and coarticulation cues while simulating realistic device, channel, noise, and reverberation variability.

Future work will aim to mitigate potential recording-condition shortcuts in the in-domain cohort by quantifying recording quality and channel artifacts (e.g., SNR/noise level, reverberation proxies, codec/compression indicators). Additionally, performing sensitivity analyses via stratification or exclusion of low-quality recordings may provide a clearer estimate of in-domain performance under varying acoustic conditions.

### Implications for digital health deployment

The pipeline presented in this study is referred to as the “nasality pretrained screening (NPS)” pipeline, which is deployment friendly given the frozen encoder and lightweight downstream classifier. This design supports real-world screening workflows, where recordings may be captured using diverse consumer devices and acoustic environments (e.g., home or web-sourced recordings), and inference can be performed either on device or in a server-assisted manner depending on resource constraints. In addition, recording-level aggregation by averaging chunk probabilities provides a simple and interpretable decision mechanism that can be used consistently across settings, supporting a screening workflow where repeated measurements may be collected longitudinally. This deployment-oriented framing is aligned with broader efforts in voice-based digital biomarkers and scalable screening pipelines beyond VPD (38).

### Limitations

The proposed method has several drawbacks and limitations that should be acknowledged, along with corresponding directions for mitigation. First, the nasality pre-training supervision is derived from phoneme-alignment context rules (oral vs. nasal neighboring consonants). Because this rule-based labeling provides only an approximate proxy for nasality and may be noisy or incomplete, the resulting pretrained representation may not capture all clinically relevant variability in co-articulation, speaking style, and severity-dependent acoustic patterns. Relatedly, the same-speaker and same-vowel positive-pair sampling strategy was intentionally conservative: it reduces speaker and phonetic-content confounding under proxy supervision, but it may not fully enforce cross-speaker or cross-vowel invariance. Incorporating cross-speaker or cross-vowel positive pairs may improve invariant representation learning in larger or bettercontrolled datasets, but doing so with noisy context-derived labels could also introduce false-positive pairs by forcing acoustically heterogeneous samples to be treated as equivalent. Future work should investigate curriculum-based or hierarchical contrastive objectives that gradually incorporate cross-speaker and crossvowel positives while preserving nasality-specific discrimination. In addition, no acoustic degradation augmentation was applied during nasality SupCon pre-training. Although this design helped isolate the effect of the nasality-focused contrastive objective, it may limit robustness to device, channel, noise, and reverberation variability. Future work should evaluate nasality-preserving augmentation strategies, including simulated room acoustics, microphone/channel filtering, background noise, and other real-world recording degradations, while ensuring that such transformations do not obscure or distort nasality-relevant speech cues. Second, the OOD dataset may contain uncontrolled confounders (device, environment, demographics, compression, and recording protocols), and the heterogeneous public Internet sources provide limited metadata, which limits our ability to derive causal attribution of the performance changes to any single factor (6). However, the extreme variability of the OOD cohort likely exaggerates what would be encountered in the field, which may underestimate model performance. Additionally, since reliable speaker identifiers are unavailable in the public sources, we treat each recording as an independent evaluation unit. Yet this recording-level assumption may be violated if some recordings are correlated (e.g., multiple clips from the same speaker). As such, future investigations should validate robustness under speaker-disjoint OOD designs when identifiers are available. Third, although the in-domain clinical cohort remains modest in absolute size for machine learning, it is comparatively large for AI-based VPD screening and uses dual CCC-SLP-verified binary labels with perfect inter-rater agreement (Cohen's *κ* = 1*.*0). Nevertheless, the added subject-level nested 5-fold cross-validation analysis showed nonzero fold-to-fold variation, indicating that performance estimates remain sensitive to cohort composition. In addition, near-ceiling performance under standardized in-domain recording conditions may still overestimate robustness in broader populations, different elicitation prompts, or alternative clinical workflows. Moreover, the in-domain case and control groups may differ in demographic characteristics (e.g., age distributions), which could be a significant confounding factor and should be addressed in larger matched cohorts. Finally, a fixed screening threshold (*t* = 0*.*5) was used for simplicity and comparability across methods. In practice, the operating point may need to be tuned to clinical priorities (e.g., prioritizing sensitivity for screening) and probability calibration may be required across deployment settings.

### Future directions

Next steps include collecting larger age- and sex-matched multi-device datasets with standardized clinical metadata to better characterize which recording-condition factors (microphone response, room acoustics, noise, and compression) drive generalization failures, and to support principled robustness evaluations under controlled perturbations. These data would also enable more reliable nasality-preserving augmentation, cross-speaker and cross-vowel contrastive sampling, demographic-confounding analysis, and deployment-aware probability calibration. Additionally, the screening task will be extended beyond binary detection toward severity grading and longitudinal monitoring of treatment response. Methodologically, exploring domain-robust objectives (e.g., domain-adversarial learning or correlation-alignment style regularization) may further reduce sensitivity to recording artifacts (7, 8). Finally, motivated by deployment in resource-limited and multilingual settings, we aim to study cross-lingual generalization and multilingual VPD screening, with the goal of developing models that maintain strong diagnostic performance across languages such as English and Spanish without requiring extensive language-specific re-collection or re-training.

## Conclusion

This study presents a nasality-focused representation learning framework to improve the robustness of model-based VPD screening under substantial recording-domain shift. Using supervised contrastive pretraining with oral-context versus nasal-context supervision, the proposed encoder learns nasality-relevant cues while suppressing speaker- and phonetic-content confounds, enabling a frozen feature extractor with lightweight downstream classifiers for recording-level screening. Under standardized in-domain clinical recording conditions, performance was near ceiling on the subject-disjoint held-out test set, and an additional subject-level nested 5-fold cross-validation sensitivity analysis showed consistently strong in-domain performance when only the second-stage classifier was retrained. More importantly for deployment, the proposed approach improved OOD screening performance on heterogeneous public Internet recordings without retraining or calibration, outperforming the strongest retained baseline under the same evaluation protocol and fixed threshold. Overall, these results suggest that explicitly targeting physiologically meaningful attributes at the representation level can reduce reliance on recording artifacts and support deployable speech-based screening in real-world settings. Future work will prioritize prospective multi-device data collection with standardized metadata and deployment-aware calibration to establish clinically robust operating points.

## Data Availability

The in-domain clinical recordings were collected and curated through the Department of Plastic Surgery at Vanderbilt University Medical Center. Due to privacy, ethical, and institutional restrictions, these clinical data are not publicly available. Requests for access may be considered upon reasonable request to the corresponding author and subject to applicable institutional approvals and data use requirements. The out-of-domain test audio samples were obtained from publicly available Internet sources; control samples were obtained from the Centers for Disease Control and Prevention (CDC Milestones in Action: https://www.cdc.gov/act-early/milestones-in-action/index.html) and the Eastern Ontario Health Unit (Eastern Ontario Health Unit Let's Talk video: https://www.youtube.com/watch?v=K0aHjxzDb7I).
